# Paediatric Common Infections Pathways: improving antimicrobial stewardship and promoting ambulation for children presenting with common infections to hospitals in the UK and Ireland

**DOI:** 10.1093/jacamr/dlab029

**Published:** 2021-03-12

**Authors:** Carolyne Horner, Robert Cunney, Alicia Demirjian, Conor Doherty, Helen Green, Mathew Mathai, Paddy McMaster, Alasdair Munro, Stéphane Paulus, Damian Roland, Sanjay Patel

**Affiliations:** 1 The British Society for Antimicrobial Chemotherapy, Birmingham, UK; 2 Temple Street Children's University Hospital, Dublin, Ireland; 3 Evelina London Children's Hospital, London, UK; 4 Royal Hospital for Children, Glasgow, UK; 5 Southampton Children’s Hospital, University Hospital Southampton NHS Foundation Trust, Southampton, UK; 6 Bradford Teaching Hospitals NHS Trust, Bradford, UK; 7 North Manchester General Hospital, Manchester, UK; 8 Children’s Hospital, Oxford University Hospitals NHS Foundation Trust, Oxford, UK; 9 University of Leicester NHS Trust, Leicester, UK

## Abstract

Paediatric common infection pathways have been developed in collaboration between the BSAC and national paediatric groups, addressing the management of cellulitis, lymphadenitis/lymph node abscess, pneumonia/pleural empyema, pyelonephritis, tonsillitis/peritonsillar abscess, otitis media/mastoiditis, pre-septal/post-septal (orbital) cellulitis, and meningitis. Guidance for the management of a child presenting with a petechial/purpuric rash and the infant under 3 months of age with fever is also provided. The aim of these pathways is to support the delivery of high-quality infection management in children presenting to a hospital. The pathways focus on diagnostic approaches, including the recognition of red flags suggesting complex or severe infection requiring urgent intervention, approaches to antimicrobial stewardship (AMS) principles and guidance on safe and timely ambulation aligned with good practice of outpatient parenteral antimicrobial therapy (OPAT).

## Introduction

Antimicrobial resistance (AMR) is an urgent and increasing threat, especially in children.^1–5^ In the UK, approximately 41% of hospitalized children receive at least one antimicrobial agent during their admission.^6^ Although antimicrobial stewardship (AMS) programmes have been implemented in the majority of children’s hospitals in the UK,^7^ very little emphasis has been placed on paediatric antimicrobial stewardship in secondary care settings, despite most children receiving intravenous (IV) antibiotics in the UK being managed in secondary care centres. As such the quality of AMS for children is extremely variable.^7^

Most patients in the UK (adults and children) are seen in district general hospitals, not teaching hospitals/tertiary centres. Whilst most district general hospitals offer adult infection services (infectious diseases and/or clinical microbiology), almost no district general hospitals have specialists in paediatric infectious diseases or paediatricians with a specific interest in infection. Currently, most paediatricians refer to their local antibiotic guidelines, where they exist, but management can differ between clinicians in the same team, within teams in hospitals, and between local hospitals (authors’ experience). This may lead to confusion within teams and ultimately patient risk.

There is an increasing evidence base supporting the use of shorter parenteral antimicrobial courses for numerous pathologies in adults. Evidence from the paediatric literature indicates that for children with severe infections who initially received IV antibiotics, earlier IV to oral switches and shorter total durations of antibiotic therapy are associated with equivalent outcomes, assuming the child can tolerate/absorb oral antimicrobials and adherence to oral treatment regimens without regular oversight is not a concern.^8^^,^^9^

Embedding paediatric antimicrobial stewardship principles within general paediatric services has been shown to safely reduce the duration of IV antimicrobials through earlier cessation of antimicrobial therapy or switching from IV to oral agents, compared with children being managed outside of services with paediatric AMS oversight.^10–13^ This is especially relevant when children are being ambulated (allowed to return home and return to visit the hospital on a regular basis to receive IV antibiotics) directly from the emergency department or paediatric assessment unit as part of an admission-avoidance strategy.^14^ In the absence of paediatric AMS oversight, children have higher rates of ‘bug–drug’ mismatches, drug-dosing errors, readmission rates and less-rigorous laboratory monitoring of drug side effects.^15^^,^^16^

The aim of this collaboration was to develop pathways for the management of children (<18 years old) presenting to district general hospitals with common community-acquired infections, including cellulitis, lymphadenitis/lymph node abscess, pneumonia/pleural empyema, pyelonephritis, tonsillitis/peritonsillar abscess, otitis media/mastoiditis, pre-septal/post-septal (orbital) cellulitis, and meningitis. Pathways for the management of children presenting to hospital with petechial/purpuric rashes and for infants under 3 months of age presenting with fever were also developed.

The aim of this article is to inform how the pathways were created. We present the rationale behind the development of the pathways, including details of the robust methodology applied to inform each pathway, collaboration with specialist groups, and consultation process with wider stakeholders. Plans for widespread implementation of the pathways are also included.

## Methods

### Scope and purpose

**Figure 1.**
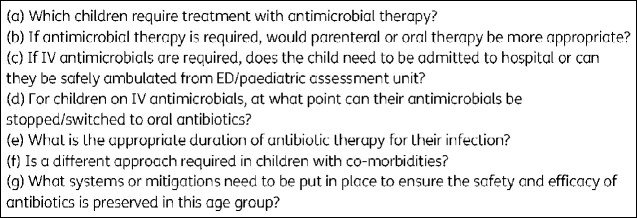
Questions answered within each pathway.

The rationale for these pathways was to support the delivery of high-quality infection management in children presenting to hospital, focusing on diagnostic approaches including the recognition of red flags suggesting complex or severe infection requiring urgent intervention, approaches to management adhering to the principles of AMS and guidance on safe and timely ambulation aligned with the principles of outpatient parenteral antimicrobial therapy (OPAT). Review of the current evidence on antimicrobial prescribing in children and areas for improvement were used to derive a list of questions to be answered within each pathway (Figure 1).^13–15^

Although the aim of the pathways was to provide guidance aligned with the principles of AMS, recommendations were not made on the choice of antimicrobial agent(s) within these pathways. This approach was taken because deciding on choice of antimicrobial agent requires local discussions with key stakeholders along with review of local antimicrobial resistance rates; the Pathway Working Party were concerned that offering guidance on choice of antimicrobial agent may be challenged by local clinicians, leading to unnecessary variability in care and potentially missed opportunities for optimal care delivery. The pathways signpost users to the recently developed National Institute for Health and Care Excellence (NICE) guidelines for common infections, where applicable.^17^

The pathways are aimed at paediatricians, emergency department clinicians, advanced paediatric nurse practitioners, microbiologists, and pharmacists.

### Data sources and search strategy

The Cochrane Database of Systematic Reviews (Issue 10 of 12, October 2019), CINAHL, EMBASE and MEDLINE databases were comprehensively searched from 1 January 2014 to 16 October 2019 using the search criteria of McMullan *et al.* (2016)^8^ (Table S1, available as Supplementary data at *JAC-AMR* Online). The same databases were searched using criteria associated with paediatric OPAT (pOPAT) (as published previously^18^) from 1 January 2018 to 16 October 2019. Studies were included for review if they included children (<18 years old) presenting with the infections of interest to secondary care settings. Articles published in full in peer-reviewed English language journals were acceptable; those in journal supplements were considered. Studies were excluded if they comprised exclusively adult patients (≥18 years old), or infections managed exclusively in primary care or exclusively in tertiary centres. Studies comprising paediatric patients with cystic fibrosis, bronchiectasis, or post-operative infections were also excluded. Full details of the inclusion and exclusion criteria applied are listed in Table S2.

### Literature review

**Figure 2.**
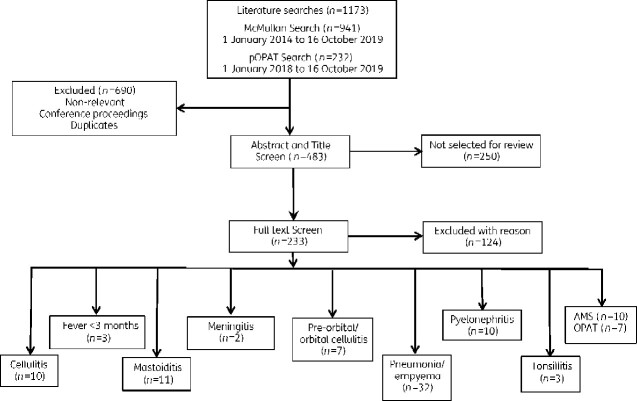
Flow diagram illustrating the process of the literature search and selection. No studies were identified for lymphadenitis or petechial rash. Some studies may have covered more than one topic.

A total of 1173 references were identified from the literature search (Figure 2): 941 references based on the McMullan *et al*.^8^ search strategy and 232 references using the pOPAT search strategy. An initial screen identified non-relevant references (*n = *690) (see Table S2 for how non-relevant references were identified), which were removed from further appraisal. An initial selection of eligible references (*n = *483) was completed by two individuals by screening titles and abstracts. There were 233 references selected for further appraisal and the full articles available in English were reviewed. References from the literature search considered to be of relevance (*n = *109) were categorized according to pathway (Figure 2). Additional references were identified by the authors who prepared each pathway. The references used to inform each pathway are cited.

### Collaborative approach to pathway development

The British Society for Antimicrobial Chemotherapy (BSAC) was the host organization. Working Party membership comprised consultants in paediatric medicine, paediatric emergency medicine, ambulatory care, paediatric infectious diseases, and medical microbiology, an antimicrobial pharmacist, and a clinical nurse specialist in paediatric infectious diseases. Pathways were drafted in partnership with national professional groups [British Paediatric Allergy Immunology & Infection Group (BPAIIG), Association of Paediatric Emergency Medicine (APEM), British Association for Paediatric Otolaryngology (BAPO), ENT UK, British & Irish Paediatric Ophthalmology and Strabismus Association (BIPOSA) and the British Paediatric Respiratory Society (BPRS)].

### Consultation process

Draft pathways were circulated to all authors and speciality groups for agreement before wider consultation. Representatives from all speciality groups provided detailed feedback regarding their relevant pathway (comments not shown) and each pathway was revised before wider consultation. To gather feedback and improve the quality of the content of the pathways, the draft pathways were sent to a comprehensive list of stakeholders (Table S3), uploaded to the BSAC website (www.bsac.org.uk), and a formal 2 week consultation process was completed (19/10/20–02/11/20). The pathways were revised in light of the 151 comments received (Table S4).

### Pathways

Ten pathways were created. Pathways for the management of children presenting to hospital with common community-acquired infections including cellulitis, lymphadenitis/lymph node abscess, pneumonia/pleural empyema, pyelonephritis, tonsillitis/peritonsillar abscess, otitis media/mastoiditis, pre-septal/post-septal (orbital) cellulitis, meningitis, management of children presenting to hospital with petechial/purpuric rashes, and infants under 3 months of age presenting with fever, are freely available online at: https://bsac.org.uk/paediatricpathways/.

Each pathway comprises sections on diagnosis, including differential diagnoses, necessary investigations, and guidance on how to assess severity (e.g. red flags) that may indicate severe/complex infection and a need for urgent intervention. Management options for mild, moderate, and severe infection are presented, including when antibiotics (IV or oral) are required. Although the specific choice of antimicrobial agent was not included, criteria for IV-to-oral switch and total duration of antibiotic course were provided, as was guidance for patient admission or ambulation. Indications for source control/surgical management were included, as was the need for ongoing monitoring, follow-up, and further investigations. Links to national guidance and safety-netting advice were provided.

## Discussion

The impact of AMR on children is being increasingly recognized, and local systems, processes and pathways need to be introduced to reduce the unnecessary variability of antimicrobial prescribing in children. This piece of work is based on the large body of evidence supporting the role of empirical antimicrobial guidelines and clinical pathways in improving the quality of antibiotic prescribing and antimicrobial stewardship.^19–21^

However, although NICE provides guidance on the choice of antibiotic for managing common infections in children,^17^ there remains a paucity of evidence-based guidance focusing on the principles of AMS and ambulation in children presenting to hospital. This results in a significant proportion of children being treated with broad-spectrum antibiotics while remaining in hospital for longer than necessary.^10^ This is costly for hospitals and the health system, inconvenient for families and contributes to the problem of antimicrobial resistance. Hence, there is an urgent need for paediatric-specific pathways addressing common infectious conditions.

The pathways presented here provide a practical approach to antimicrobial management in children presenting to hospital, including clear guidance on when children can be safety ambulated and the clinical governance systems required to facilitate this. The strength of these pathways lies in the robust scientific process adopted to identify relevant literature, in addition to collaboration with relevant national bodies to develop each pathway. In order to avoid overlap with NICE guidance, the pathways signpost readers to guidance on choice of antibiotic where it is available, such as guidance provided by the UK Paediatric Antimicrobial Stewardship (UK PAS) Network (https://www.uk-pas.co.uk/).

Data support the availability of online access to improve dissemination of clinical pathways.^22^ All pathways are hosted online and are optimized for use on a mobile device. Plans for implementation of these pathways include dissemination via national speciality and professional groups, via social media, and the development of educational packs to facilitate locally delivered teaching, including education by PAS teams during regional teaching. In addition, e-learning resources are being produced to support self-directed learning.

In the short term, it is hoped that successful implementation of these pathways will result in reduced variability in care and ultimately improve the clinical management of children presenting to district general hospitals, which in many cases will result in shortened duration of antibiotic courses, earlier transition from IV to oral therapy and timely ambulation from hospital. We hope that in the longer term, these pathways will contribute to a reduction of the development of antimicrobial resistance in children.

## Supplementary Material

dlab029_Supplementary_data
