# Supplementary material for: Paediatric Common Infections Pathways: improving antimicrobial stewardship and promoting ambulation for children presenting with common infections to hospitals in the UK and Ireland
Source: JAC Antimicrob Resist. 2021 Mar 12;3(1):dlab029. doi: 10.1093/jacamr/dlab029 (PMC8210287; doi:10.1093/jacamr/dlab029)
Supplement: dlab029_Supplementary_data [file dlab029_supplementary_data.docx]

**Supplementary data**

**Table S1:** Search criteria used for evidence identification

**Table S2**: Evidence selection criteria

**Table S3:** Consultation Stakeholders

**Table S4:** Consultation responses received by the end of a formal consultation process

***Table S1:*** *Search criteria used for evidence identification*

| **Search No.** | **Date** | **Databases searched**  **(limits: 2014-present; English language)** | **Search results (before duplicate removal)** |
| --- | --- | --- | --- |
| 1 | 16/10/2019 | Medline (OVID) | 451 |
| 2 | 16/10/2019 | Embase (OVID) | 478 |
| 3 | 16/10/2019 | Cinahl (EBSCOHost) | 58 |
| 4 | 16/10/2019 | Cochrane Library issue 10 2019 | 123 |
| Total number results | | | 1110 |
| Total number results in Endnote after removing duplicates | | | 941 |

**Ovid MEDLINE(R) and Epub Ahead of Print, In-Process & Other Non-Indexed Citations, Daily and Versions(R) <1946 to October 15, 2019>**

Indexed Citations, Daily and Versions(R) <1946 to October 15, 2019>

1 exp Anti-Bacterial Agents/

2 exp Administration, Oral/

3 (oral$ or per os or po).tw.

4 2 or 3

5 Infusions, Intravenous/

6 Injections, Intravenous/

7 (intra-venous$ or intravenous$ or iv or parenteral$).tw.

8 5 or 6 or 7

9 1 and 4 and 8

10 Time Factors/

11 ((short* or long*) adj3 (length or period or duration)).tw.

12 ((one or two or three or four or five or six or seven or eight or nine or ten) adj (day or days)).tw.

13 (("1" or "2" or "3" or "4" or "5" or "6" or "7" or "8" or "9" or "10") adj (day or days)).tw.

14 ((one or "1" or two or "2") adj (week or weeks)).tw.

15 11 or 12 or 13 or 14

16 1 and 10

17 1 and 15

18 16 or 17

19 exp Infant/ or exp Child/ or Adolescent/

20 (child* or infant* or newborn* or babies or boys or girls or adolescen* or paediatric* or pediatric*).tw.

21 19 or 20

22 9 or 18

23 21 and 22

24 limit 23 to (english language and yr="2014 -Current")

25 Cellulitis/ or cellulitis.mp.

26 Tonsillitis.mp. or Tonsillitis/

27 Otitis media.mp. or Otitis Media/

28 exp Pneumonia/

29 empyema.mp. or exp Empyema/

30 Orbital Cellulitis/ or Periorbital cellulitis.mp.

31 Pyelonephritis/ or Pyelonephritis.mp.

32 Meningitis.mp. or exp Meningitis/

33 Mastoiditis.mp. or Mastoiditis/

34 Lymphadenitis.mp. or exp Lymphadenitis/

35 Osteoarthritis/ or Osteomyelitis/ or Arthritis, Infectious/ or Osteoarticular infections.mp.

36 Petechial rashes.mp.

37 25 or 26 or 27 or 28 or 29 or 30 or 31 or 32 or 33 or 34 or 35 or 36

38 24 and 37

-End-

**Table S2**: Evidence selection criteria

| ***Inclusion criteria*** | ***Population***  Children (<18 years old) presenting with common infectious presentations to secondary care settings, including cellulitis, tonsillitis, otitis media, pneumonia (including empyema), periorbital cellulitis, pyelonephritis, meningitis, mastoiditis, lymphadenitis, petechial rashes and fever in the young infant (<3 months). |
| --- | --- |
|  | ***Setting***  Secondary care settings include emergency departments, paediatric assessment units/short stay units and in-patient setting. |
|  | ***Study types***  Randomised control trials (RCT), controlled clinical trials (CCTs), interrupted time series with at least three data points before and after implementation of the intervention (ITS), controlled before and after studies (CBA). Systematic reviews and meta-analyses, case-controlled studies, case series comprising >10 patients, and journal supplements will be considered.  Articles in English language will be included, and full journal publication was required. |
| ***Exclusion criteria*** | Adult patients (≥18 years old), infections managed exclusively in primary care or exclusively in tertiary centres, paediatric patients with cystic fibrosis, bronchiectasis, or post-operative infections. |
|  | References with no named author (blank or anon.), case reports (defined as ≤3 patients), animal studies, abstract and conference proceedings, correspondence, and in a language other than English. Other non-relevant references were defined as studies about other clinical conditions not within the scope of the pathways (such as melioidosis, tularaemia, *Burkholderia pseudomallei*), *in vitro* studies, editorials, and duplicate references. |

**Table S3:** Consultation Stakeholders

List of stakeholders to whom the pathways were sent for consultation. Those with a paediatric speciality are shown in **bold**.

|  | **Consultation Group** |
| --- | --- |
| A | Academy of Medical Royal Colleges |
|  | **Academic Paediatrics Association of Great Britain and Ireland** |
|  | Association for Nurse Prescribing |
|  | **Association of Paediatric Emergency Medicine** |
|  | Association of Surgeons of Great Britain & Ireland |
|  | Association of the British Pharmaceutical Industry |
| B | British Association for Cancer Surgery |
|  | **British Association of General Paediatrics** |
|  | **British Association of Paediatric Nephrology** |
|  | **British Association of Paediatric Surgeons** |
|  | British Association of Plastic, Reconstructive and Aesthetic Surgeons |
|  | British Cardiac Patients Association |
|  | British Cardiovascular Society |
|  | British Dental Association |
|  | British Heart Foundation |
|  | British Heart Rhythm Society (formerly Heart Rhythm UK) |
|  | British Heart Valve Society |
|  | British HIV Association |
|  | **British & Irish Paediatric Ophthalmology and Strabismus Association** |
|  | British Lung Foundation |
|  | British Medical Association |
|  | British Orthopaedic Association |
|  | **British Paediatric Allergy, Immunology & Infection Group** |
|  | British Paediatric Respiratory Society |
|  | British Pharmacological Society |
|  | **British Society for Children’s Orthopaedic Surgery** |
|  | British Society of Echocardiography |
|  | British Society for Medical Mycology |
|  | **British Society for Paediatric Gastroenterology, Hepatology and Nutrition** |
| C | Care Quality Commission |
|  | CDiff Support |
|  | Central Sterilising Club |
|  | **Children’s Cancer and Leukaemia Group** |
|  | **Children’s HIV Association** |
|  | Clinical Virology Network |
|  | Community Pharmacy Scotland (formerly Scottish Pharmaceutical General Council) |
|  | Consumer Futures (formerly the National Consumer Council) |
| **D** | **Department of Health and Children (Ireland)** |
|  | Department of Health Social Services & Public Safety (NHS Northern Ireland) |
| E | European Society of Clinical Microbiology and Infectious Disease |
| F | Faculty of Intensive Care Medicine |
|  | Faculty of Pharmaceutical Medicine |
|  | Faculty of Public Health |
| G | General Dental Council |
|  | General Medical Council |
|  | General Pharmaceutical Council |
|  | Guild of Healthcare Pharmacists |
| H | Health Protection Society |
|  | Health Protection Scotland |
|  | Healthcare Improvement Scotland (NHS) |
|  | Healthcare Infection Society |
|  | Heart Research UK |
| I | Independent Alliance of Patients and Healthcare Workers for Hand Hygiene |
|  | Infection Prevention Society |
|  | Institute of Decontamination Sciences |
|  | **Irish Paediatric Association** |
| L | Lee Spark Necrotising Fasciitis Foundation |
| M | Medical Defence Union |
|  | Medical Protection Society |
|  | Medical Research Council |
|  | Medical Schools Council |
|  | Microbiology Society (formerly Society for General Microbiology) |
|  | MRSA Action UK |
| N | National Infusion and Vascular Access Society |
|  | National Institute for Health and Clinical Excellence |
|  | National Institute for Health Research |
|  | National Pharmacy Association |
|  | **Neonatal and Paediatric Pharmacy Group** |
|  | NHS Commissioning Board Special Health Authority  (formerly the National Patient Safety Agency) |
|  | NHS Confederation |
|  | NHS England |
|  | NHS Improvement |
|  | NHS Providers |
| P | Parliamentary and Health Service Ombudsman |
|  | **Paediatric Intensive Care Society** |
|  | **Paediatric Microbiology Group** |
|  | Patients Association |
|  | Pharmaceutical Quality Group |
|  | Pharmaceutical Society of Northern Ireland |
|  | Public Health England |
|  | Public Health Wales |
| Q | Quality Improvement Scotland (NHS) |
| R | Research Quality Association  (formerly the British Association of Research Quality Assurance) |
|  | Royal College of Anaesthetists |
|  | Royal College of Emergency Medicine |
|  | Royal College of General Practitioners |
|  | Royal College of Midwives |
|  | Royal College of Nursing |
|  | Royal College of Obstetricians & Gynaecologists |
|  | Royal College of Ophthalmologists |
|  | Royal College of Pathologists |
|  | **Royal College of Paediatrics and Child Health** |
|  | Royal College of Physicians & Surgeons |
|  | Royal College of Physicians of London |
|  | Royal College of Psychiatrists |
|  | Royal College of Radiologists |
|  | Royal College of Surgeons |
|  | Royal College of Surgeons (Edinburgh) |
|  | Royal Pharmaceutical Society |
|  | Royal Society for Public Health |
|  | Royal Society of Tropical Medicine and Hygiene |
| S | Scottish Association of Health Councils |
|  | Scottish Intercollegiate Guidelines Network |
|  | Scottish Medicines Consortium |
|  | Society for Acute Medicine |
|  | Society of Critical Care Medicines |
|  | Standards for Microbiology Investigations |
|  | Surviving Sepsis Campaign |
| T | The British Society for Allergy & Clinical Immunology |
|  | The British Thoracic Society |
|  | The Consumers' Association (Which?) |
| U | UK Clinical Pharmacy Association |
| W | Welsh Assembly Government |
|  | Welsh Microbiological Association |
|  | **Welsh Paediatric Society** |

-End-

**Table S4:** Consultation responses received by the end of a formal consultation process (19.10.20 – 02.11.20) (n = 151)

| **Source** | **Pathway** | **Section** | **Comment** | **COMMITTEE RESPONSE / DECISION** |
| --- | --- | --- | --- | --- |
| Speciality Group | AOM |  | My only comment would be in AOM why we would need the box in orange below management which suggests consider IVs for mild infection where patient can't tolerate orals -seems like a rare scenario and that box could conceivably be deleted without much loss. | Agree - removed |
| Speciality Group | AOM |  | in the box comparing otitis externa to AOM there is a line about discharge. Would it be helpful to somehow make it clear that discharge doesn’t always happen with AOM, only when there is a perforation? I know that they are trying to keep it as simple as possible though. | Amended |
| Consultation | AOM | General | Layout as presented here does not allow to read all the way down to bottom of ‘severity scoring table’ - should there be any other points below ‘Eating less’ ? | Formatting will be optimised when converted to digital format |
| Consultation | AOM | Management Mild infection 6 months-2 years | … or if symptoms score >8# (slide 3) but #Severity score (green box on slide 4) max is only 8 points | In light of other comments, this severity scoring system has been removed as consultation responses suggest too complicated. |
| Consultation | AOM | General | This is just too complicated – It’s a struggle to work out what is meant by mild/moderate or severe. Would it not just be simpler to advise IV antibiotics (+/- imaging) for any patient with red flags (as per your definition), and oral for those less than 2 with bilateral disease, symptoms persisting>3 days or otorrhea? You could then give general advice about pain management and de-escalation of abx separately | Scoring system for 6 month-2 year olds removed as too complex. Recommendations for Abs under 2 years as per NICE guidelines. Separated analgesia and antibiotic recommendations as suggested. |
| Consultation | AOM |  | Layout as presented here does not allow to read all the way down to bottom of severity scoring table - should there be any other points below ‘Eating less’ ? Cannot get symptom score >8 Usually not a systemic illness causing sepsis so confusing as to whether OM or sepsis pathway in severe. | Formatting will be optimised when converted to digital format. Severity scoring system has been removed as consultations responses suggest too complicated. This is an OM AND mastoiditis pathway- and can be associated with CNS complications - this is reflected in the RED box. Although rare, Gp A strep mastoiditis can be associated with toxic shock syndrome in which urgent drainage is potentially lifesaving. |
| Consultation | AOM | Investigations>Evaluate severity | Moderate severity – seems to suggest all children with otitis media and fever are moderately severe and so have bloods taken and receive IV antibiotics. Would be helpful to give more detail in this differentiation – to prevent a lot of children getting IV antibiotics. | AGREE - amended to clarify that not just fever: "MILD = systemically well or fever but haemodynamically stable MODERATE = systemically unwell including fever AND persisting tachycardia / tachypnoea" |
| Consultation | AOM | Investigations | ?Moderate = “fever, tachycardia OR tachypnoea” [rather than AND] – however, not all those with moderate AOM need bloods. Therefore, could be less prescriptive: e.g. moderate = ‘Systemically unwell but no red flags’ | AGREE - amended to clarify that not just fever: "systemically unwell including fever AND persisting tachycardia / tachypnoea" |
| Consultation | AOM | Management>upper right | “If AOM in a child with tympanostomy tubes, treat with non-ototoxic topical Abx” is this in addition to orals? Needs clarifying. Also, needs “If requiring Abx…” in this box. | AGREE - amended |
| Consultation | AOM | Management | I would restrict to 5 days course for uncomplicated infection; Table 1 need to define how many points for mild, moderate and severe, this is somewhere in the guideline but would be helpful to have it on this table too | AGREE - amended. Scoring system for children aged 6 months-2 years removed as deemed too complicated during national consultation. |
| Consultation | AOM | Investigations | ideally collect a sample of pus using a sterile syringe -without a needle, swabs tend to have limited diagnostic value | Although this may improve ability to interpret results, this is a large step change in terms of practice and needs to be supported by national bodies since NICE before it can be added here. |
| Consultation | AOM | Management | Should say size, every attempt should be made for drainage here, if not feasible or too small, consider diagnostic aspiration, implications for 6-8weeks of Rx | Amended to size as suggested. This guidance is not aimed to provide definitive guidance for the management of intracranial abscesses. We amended "Management of Intracranial complications depends on type and requires ENT and neurosurgical input" to "requires ENT, neurosurgical and infectious diseases input" - this will ensure that appropriate discussions about sample collection and duration of Abs take place. |
| Speciality Group | AOM |  | In section ‘$ If neuro-ontological imaging performed, ENT review of imaging findings it states – “site, site…” there is duplication of the word site. | Amended to "size" |
| Speciality Group | Cellulitis |  | Is it worth including toxic shock syndrome in the differentials box? | Tends to be a generalised, non-tender, non-hot rash rather than one that mimics cellulitis. Necrotising fasciitis is included in the differential list. This comment has not been replicated in the national consultation. |
| Speciality Group | Cellulitis |  | Is it worth narrowing duration of treatment right down to 5 or 7 days? Our local Micro department have opted for 5 days for some things, and 7 for others. If we are trying to standardise use of antibiotics now might be the time to be clear about duration (my own opinion). | NICE guidelines suggest 5-7 days and no clear evidence between5 and 7 days. National empirical Ab guidelines produced by PAS-UK also suggest 5-7 days. |
| Speciality Group | Cellulitis |  | On the cellulitis one, I'm a little concerned regarding orbital cellulitis. The Melbourne scoring only gives 1 point for eye involvement, but then the yellow box says 'consider IV antibiotics' if 'facial involvement'. I'd probably count the eye as part of the face and would be a little confused whether to start oral or IV antibiotics based on this for peri-orbital cellulitis but would be concerned that orbital cellulitis could be missed completely without considering photophobia, proptosis, pain on eye movements etc.. I wonder if orbital cellulitis should be mentioned specifically as has one of the most serious complication potentials? | The cellulitis pathway is specifically not for periorbital/orbital cellulitis (we have developed a separate pathway).  Have amended title to reflect this "For periorbital/orbital cellulitis, see separate pathway" |
| Speciality Group | Cellulitis |  | I'd consider adding a caution regarding cellulitis over a joint and raise the possibility of septic arthritis/osteomyelitis, particularly in younger populations. | Agree - have added the following to the differential list "In a young infant with erythema over a joint or bone, consider septic arthritis or osteomyelitis" |
| Consultation | Cellulitis | Investigations | I would consider taking blood cultures in complicated/complex cellulitis. In our trust, we have come across cases of MRSA/MSSA and *Strep pyogenes* bacteraemias usually from patients with complex cellulitis. I agree that in uncomplicated cellulitis, investigations are usually unnecessary | Agree - have added "In children with complex cellulitis#, consider full blood count, CRP and blood culture." |
| Consultation | Cellulitis | Investigations>General | Consider Odontogram – would it be better to have consider dental review or odontogram – as need for tooth extraction could be identified without an X-ray. And access to dental review and interpretation of odontogram might depend on local services. No mention of osteomyelitis in differential  Nec-fasciitis – extremely painful on touch | AGREE- have amended to "Consider dental review and/or odontogram ". Have added "In a young infant with erythema over a joint or bone, consider septic arthritis or osteomyelitis" to differential list. Have amened to "extreme pain over site of erythema often disproportionate to the extent of the rash" in line with BMJ best practice article (referenced) |
| Consultation | Cellulitis |  | Features of complex cellulitis, I would list post chickenpox as well and put in this list a reference (dash or something to refer to the Melbourne criteria table) Management for mild infection : worsening of cellulitis in spite of adequate oral antibiotic type and DOSE  Duration 5 days | AGREE: amended to "adequate oral Abx (check dose and adherence)". Have added clarity about concerns about post-VZV cellulitis - "In addition, lower threshold for starting IVAbx if features of complex cellulitis" and "Lower threshold for admission prior to ambulation if features of complex cellulitis". Regarded 5 days - NICE guidelines suggest 5-7 days and no clear evidence between5 and 7 days. National empirical Ab guidelines produced by PAS-UK also suggest 5-7 days. |
| Consultation | Cellulitis | Management | Options for cellulitis sec to human, dog or cat bites | AGREE - have signposted to NICE guidelines (bites - human and animal bites) referenced https://www.nice.org.uk/guidance/ng184/resources/human-and-animal-bites-antimicrobial-prescribing-pdf-66142021681861 at start of pathway. |
| Speciality Group | Cellulitis |  | When suggesting doing an odontogram, the reviewer would suggest also referring to Orthodontists/MaxFax at that point. | Agree - have added |
| Consultation | Children age > 3 months with meningitis | Management>ambulation | Use of word ‘discharging’ towards end of first paragraph is confusing – I think you mean ambulating? | Agree - amended |
| Consultation | Children age > 3 months with meningitis | Confirmation>confirmed bacterial cause | ‘Send sample for confirmatory testing ‘ – needs clarification as to whether repeat test on existing sample, which is what I presume you mean, versus repeat LP | Agree - have amended as follows: "Ideally confirm biofire result by performing targeted pneumococcal PCR on existing sample." |
| Consultation | Children age > 3 months with meningitis | Management>ambulation | Use of word ‘discharging’ towards end of first paragraph is confusing – I think you mean ambulating? | Agree - amended |
| Consultation | Children age > 3 months with meningitis | Management | Duration of tx: N meningitides 5 days, P pneumo 10 days and H flu 7 days | Have aligned with national empirical Ab guidelines produced by PAS-UK: N. meningitidis 7 days S. pneumoniae 10-14 days H. influenzae (capsulate) 7-10 days E. coli 21 days |
| Speciality Group | Fever <3 months |  | only the same comment about choice of CRP cut off of 20. | Based on the STEP-BY-STEP study (See reference) |
| Speciality Group | Fever <3 months |  | Febrile infant - ?axillary temp is this the best and only way, typo in yellow box “antibiotics” | Amended |
| Consultation | Fever <3 months | General | Layout of flow sheet a bit confusing. Investigation box defines when LP should be done, but if initial investigations put infant in moderate category on basis of CRP, LP then gets added later. Most people would follow this fine, but potential for it to be missed e.g. handover between different members of staff, or someone gets distracted when consulting it? Perhaps subtle change of wording investigations column would sort this out | Agree - have amended investigations box as follows "Empirical lumbar puncture (LP) if <28 days or clinical concerns for bacterial meningitis – check for contraindications to LP" |
| Consultation | Fever <3 months | General | Layout of flow sheet a bit confusing. Investigation box defines when LP should be done, but if initial investigations put infant in moderate category on basis of CRP, LP then gets added later. Most people would follow this fine, but potential for it to be missed e.g. handover between different members of staff? Perhaps subtle change of wording investigations column | Agree - have amended investigations box as follows "Empirical lumbar puncture (LP) if <28 days or clinical concerns for bacterial meningitis – check for contraindications to LP" |
| Consultation | Fever <3 months | Management>Moderate risk | Should there also be mention of LP to be added if not done initially as not perceived to be irritable, but subsequent positive blood culture?  Mention prematurity re risk factor.  What about bloody taps (very common) and how these are interpreted re cell count? | All babies under 28 days with fever will have an LP, irrespective of B/C result. If >28 days with +ve B/C but a focus (i.e. upper UTI but no ongoing concerns about meningitis, we are not mandating an LP. Have added "Perform LP if positive blood culture (non-contaminant) in absence of focus of infection." to yellow and red boxes. Interpretation of bloody tap needs to be done on a case-by-case basis. Unable to offer useful guidance on an empirical pathway. |
| Consultation | Fever <3 months | Investigations>General | “rectal swab for enterovirus and parechovirus” – would be good to have an indication about how this might affect subsequent management. E.g. does the identification on rectal swab allow antibiotics to be stopped sooner? | AGREE - have added following to amber management box "At 36-48 hours, review clinical progression, serial inflammatory markers, microbiology and virology results as per principles of antimicrobial stewardship. If low index of suspicious for invasive bacterial infection, stop Abs" |
| Consultation | Fever <3 months | Assessing severity>high risk | Congenital malformations – would be good to have examples of those that increase risk as not all congenital malformations will increase risk | Have removed congenital malformations as very hard to align these with specific increased risk of invasive bacterial infections |
| Consultation | Fever <3 months | Investigations>HSV Comment | When would you consider HSV swabs? – I can see this leading to many neonates having these without clearer guidance, e.g. if history of maternal oral/genital herpes | Consider HSV swabs in "In babies under 4 weeks of age with fever, (collect eye/rectal/throat swabs +- blood +- CSF for HSV PCR). Only treat with empirical acyclovir awaiting results if "if ≤28 days and requiring fluid boluses or CSF pleocytosis" |
| Consultation | Fever <3 months | General | Would it be good to cover “Fever WITH source” too, or is this adequately covered by NICE Feverish illness in under 5s? | This has been covered for upper UTI. NICE are in the process of drafting a late onset sepsis guideline which will cover this. |
| Consultation | Fever <3 months | General | Perhaps note this doesn’t apply to EARLY ONSET SEPSIS, which we do occasionally see in the ED | AGREE - although the principles of management apply as per this pathway, have added a comment regarding choice of antibiotics "(use early onset sepsis guidelines if age <72 hours)". Also amended title to make it clear that this pathway does not apply to babies already on a neonatal unit "INFANT <90 DAYS OF AGE WITH FEVER AND NO SOURCE PATHWAY FOR CHILDREN PRESENTING TO HOSPITAL FROM THE COMMUNITY" |
| Consultation | Fever <3 months | Differential | add parechovirus | AGREE - added |
| Consultation | Fever <3 months | Investigations | “Consider LP” I would not add the if <28 days or if clinical concern etc. as I would be encouraging LPs in all these babies if no clear focus Assessing severity inflammatory markers, I would add WCC and PTC High risk prematurity as well | This has been discussed within the steering group and agreed to continue using the current wording. Based on the evidence, we are not advocating that all babies under 3 months of age with fever get an LP. This is in line with current NICE guidance. |
| Consultation | Fever <3 months | Management | Febrile infant guidance – can we discharge children from ED (the 1-3 month group that appear well and have normal bloods?  - if so any guidance on minimal observation periods? | AGREE that guidance on observation would be useful. Although no good evidence, pragmatic approach has been added "If the child remains clinically well after 4 hours, consider ambulating with written safety net advice and clear plans for follow up if no social risk factors" |
| Speciality Group | Fever <3 months |  | ‘Axillary temperature >38 degrees’ - In hospital or at home? This should be specified. | AGREE: if properly measured temperature of 38 degrees or above, would trigger pathway irrespective of measurement in hospital or home. This is in line with NICE guidance NG143: Subjective detection of fever by parents and carers  "Reported parental perception of a fever should be considered valid and taken seriously by healthcare professionals". Have amended as follows "Temperature ≥38°C measured by axillary thermometer (in hospital or at home)" |
| Speciality Group | Fever <3 months |  | Threshold for WCC count as well as CRP should be added especially if it presents early, a delay in CRP rise would be expected. | RESPONSE: there is no compelling evidence to support added value of WCC over CRP (although theoretically WCC rises more quickly than CRP, there are multiple studies showing that has very poor predictive value in sepsis and will lead to a large number of infants unnecessarily receiving IVAbs. This is likely to be similar for the neutrophil count). This is in line with the most recent evidence from STEP BY STEP trial (Gomez B, Mintegi S, Bressan S et al. Validation of the "Step-by-Step" Approach in the Management of Young Febrile Infants. Pediatrics 2016; 138.). We have included the importance of safety netting in this pathway.  Additional comment: I do have data on WCC and CRP in young infants. Some of it has been published also. The bottom line is that CRP is no better than PCT with a cut-off of around 20mg/l offering an optimal balance between sensitivity and specificity. In terms of WCC the overall predictive value is pretty poor and adding it in will only reduce the specificity of the guidance. I am currently running a PERUKI sponsored study specifically looking at the management of febrile infants under 90 days of age but we are unlikely to report any findings until early next year. One option is that we externally validate this guideline once it has been published." |
| Consultation | 1 | Fever <3 months | the first column states that consider observation for babies with fever 24-48hrs after imms. However that box leads an arrow to the investigation box which recommends taking bloods. I think there should be a separate box specifically for the babies post imms to emphasise observation alone otherwise may appear confusing. | No change to pathway made. To some extent, these pathways are simple guidance for clinicians - the wording is clear "•If within 24 – 48hrs of 8 week immunisations and appears well, consider period of observation +/- urinalysis if systemically well" and nobody else has raised this. We cannot start adding new boxes as will have a negative impact of the pathway overall. |
| Consultation | Fever with rash | General | Viral aetiology mentioned in differential diagnosis box but flowsheet has nothing other than ‘potential evolving meningococcal disease/DIC’ and ‘clear mechanical cause’. Surely ‘well child, petechiae only, no purpura’ should be in the lower part of the pathway | RESPONSE: the proposed approach is not in keeping with current practice or evidence (See https://www.thelancet.com/journals/laninf/article/PIIS1473-3099(20)30474-6/fulltext?rss=yes). . Unfortunately, a viral aetiology cannot be established at the point of presentation. In the absence of a clear mechanical cause, blood tests are required to decide if empirical Abs are required. This may change if further research studies are conducted. |
| Consultation | Fever with rash | Investigations>clear mechanical cause | Clotting not necessary in those with clear mechanical cause, or well child likely viral. | Response - no bloods required in the well child if clear mechanical cause. In the unwell child or child with evolving purpura, clotting is recommended. Have removed from the no-red flag child as no primary coagulopathy cases in this group in PiC study (data from Tom Waterfield). |
| Consultation | Fever with rash | General | Viral aetiology mentioned in differential diagnosis box but flowsheet has nothing other than ‘potential evolving meningococcal disease/DIC’ and ‘clear mechanical cause’. Surely ‘well child, petechiae only, no purpura’ should be in the lower part of the pathway with ‘clear mechanical cause’? In the well child with no clear mechanical cause, the following wording has been used "Blood Tests may include FBC, U&Es, Clotting, CRP, Blood Culture" | RESPONSE: the proposed approach is not in keeping with current practice or evidence (https://www.thelancet.com/journals/laninf/article/PIIS1473-3099(20)30474-6/fulltext?rss=yes) Unfortunately, a viral aetiology cannot be established at the point of presentation. In the absence of a clear mechanical cause, blood tests are required to decide if empirical Abs are required. This may change if further research studies are conducted. |
| Consultation | Fever with rash | Investigations>clear mechanical cause | Clotting should not be necessary in those with clear mechanical cause, or well child likely viral. | Response - no bloods required in the well child if clear mechanical cause. In the unwell child or child with evolving purpura, clotting is recommended. |
| Consultation | Fever with rash | Presenting features | Fever mentioned in title, but not in presenting feature. Is fever a requirement to follow this pathway. “Non blanching rash (any size or formation)” – would be good to further define this – does it mean a single petechial spot? We certainly would not pursue detailed investigation if that was the case | RESPONSE: fever is a requirement of this pathway (as per the petechiae in children study https://www.thelancet.com/journals/laninf/article/PIIS1473-3099(20)30474-6/fulltext?rss=yes) as petechiae in the absence of fever do not have the risk of meningococcal disease and the need for antibiotics this guidance is attempting to rationalise. We acknowledge the point regarding size but there will always need to be clinical discretion and size of petechiae has not yet been shown to have a negative predictive value. While it is possible this guidance may result in some unncessary treatment this is likely to be very limited given that the majority of clinicians fail to follow guidance in this area (as demonstrated by the PIC study) |
| Consultation | Fever with rash | Investigations | ?throat swab; ?rectal swab for enterovirus? | AGREE - have added the following "Virological investigations including throat swab and stool/rectal swab for enterovirus / parechovirus." |
| Consultation | Fever with rash | Investigations | “may include” is a little vague. Should this be “Blood tests, including…” | Response: clear recommendations have been included for the unwell child. For the child with no risk factors, clinical judgement should be applied to decide which investigations are appropriate. |
| Consultation | Fever with rash | Differentials | HSP is now called IgA Vasculitis (IgAV) | Have amended to HSP / IgA vasculitis as many people still use the previous terminology |
| Consultation | Fever with rash | Management>Bottom box | Home “if no other reason for admission”…. [e.g. may be vomiting excessively | AGREE - amended |
| Consultation | Fever with rash | Management>Bottom box | So if you are well, with a viral infection but a CRP of 25, it’s not totally clear if you should be getting IVAbx, or at least considering them. We do not automatically elect to give antibiotics if that is the case but would observe the child instead | RESPONSE: the Petechial rash in children trial (2020) suggests IVAbs if petechial rash and no alternative diagnosis. Have made this more clear in the management box "Otherwise commence empirical IVAbx." |
| Consultation | Fever with rash |  | Paper is to be published in Lancet Infectious Diseases (from author T Waterfield) | Have been in communication with Tom Waterfield during the development of this pathway. Paper published 10/11/20 - have added |
| Consultation | Fever with rash | Management | Public health notification as indicated, prophylaxis of close contacts | AGREE - have added to red and amber management boxes |
| Consultation | Fever with rash |  | Question from DR: If you were going to choose something to say about bloods what would it be? Would it be better just to say treat if CRP > 20 and ignore WCC?  Answer from TW: 100% Drop the wcc/neuts other than looking for haematological malignancies etc. CRP was far more useful. | AMENDED as per comment |
| Consultation | Fever with rash |  | Red Flags – Please can you clarify haemodynamically unstable? Does this mean hypotension/Tachycardia/Prolonged CRT? If so all/any | We have amended the signs of shock to marry with PIC study. However do not wish this point to distract from the need to treat those children who appear unwell. We are cautious that while PIC did reveal some characteristics predictive of meningococcal disease it will be difficult to advice not to treat in the absence of these symptoms if the clinician feels that the child appears unwell. |
| Consultation | Fever with rash |  | Rash developing – Please clarify – does that mean any spread or change at all? | Amended to spread in the department |
| Consultation | Fever with rash |  | Investigations – No child in PiC had a primary coagulopathy consider removing from the no red flags group, if sending culture I would always send PCT (1/2 PiC MD were culture negative) | Have removed clotting from non-red flag group. PCT not available in most hospitals as per a recent (unpublished) PERUKI study. It is not the remit of this group to push for test changes but NICE may well consider this. |
| Consultation | Fever with rash |  | Please clarify Abnormal Bloods – If this incudes elevated WCC/Neutrophils then this guidance will have a low specificity | This has now been amended to highlight the poor predictive value of WCC. |
| Consultation | Fever with rash |  | Management  -  Both of the children with missed MD in PiC deteriorated at around 18 hours so the 4-6 hour period would not have been useful -  I think keeping them for 4 hours is unnecessary to be honest. It’s the advice that really matters. | This has now been removed and a sentence on ensuring parental understanding has been added. |
| Speciality Group | Petechial rash |  | I'm just wondering where the CRP cut off of 20 comes from? It seems a little bit low for me to ​assume bacterial infection and give antibiotics. | From "Petechiae in children" study led by Tom Waterfield (https://www.thelancet.com/journals/laninf/article/PIIS1473-3099(20)30474-6/fulltext?rss=yes). CRP recommendation following personal communication with Tom Waterfield based on recruited cases. |
| Speciality Group | Petechial rash |  | I'm not sure how useful a U&E is in a child with a petechial rash that you are effectively screening. Fair enough in septicaemia pathway. | Amended |
| Speciality Group | Petechial rash |  | Pathways are generally really clear, and pragmatic. My only comment on the petechial rash pathway would just be around the wording for Ix in the well child. Perhaps it could say 'may include' rather than including, as I can foresee lots of well children with normal bloods having cultures and other Ix sent, who wouldn't necessarily need them. 'May include' gives the clinician a bit of flexibility. Hope that makes sense! | Amended |
| Speciality Group | Petechial rash |  | Petechiae- Should viral illness be in differential, What is “IT” next to ALL (perhaps I am tired and not thinking! ?ITP). AMENDED | Amended |
| Speciality Group | Petechial rash |  | Although ITP and HSP are mentioned in the differentials box, I'm not quite clear where they fall in the non-blanching rash guideline. Seems to me if you have purpura over 2mm you're by definition definitely getting IV antibiotics, meningococcal PCRs etc even if you clearly have ITP/HSP after a mild viral infection and your obs are completely stable. I also don't find it clear what it means by 'consider HSP'. That's still in the box which leads to all the investigations and then to IV antibiotics. Obviously post PIC study etc we have to be very careful with purpura but I'm worried this makes it a bit confusing. Could maybe by stratified by having a different box saying "purpura over 2mm but high suspicion of ITP/HSP, non-spreading rash and normal observations" or something like that? | Have amended the box to imply that a senior must sign off on not treating with Absx if HSP considered in the child with no fever (although the pathway if for the child with fever and a petechial/purpuric rash) and well appearing. Most clinicians would treat a purpuric rash with fever in the early stages even if HSP was likely. |
| Speciality Group | Formatting |  | I know these are only drafts, but I think they might look slightly more official if they weren't in primary colours and most of the boxes lined up a bit more consistently? | Formatting will be optimised when converted to digital format |
| Consultation | Formatting |  | Sorry couldn't find the form but wanted to say they look great, I like colour coding. The arrows are perhaps a bit heavy! I think it would be great to be able to link to our local abx guidelines from the document if we were to use. I have forwarded  to a paediatric consultant here to look at the clinical content. | Replied to explain that the formatting would be sorted out in the final html version |
| Consultation | General |  | Not a direct comment on the content of the pathways; I’m a GP - but I wonder if some of them & some of the content could also be used in primary care? Some are not for general practice - e.g. meningitis - but the AOM, CAP, periorbital cellulitis, UTI/pyelonephritis could be really helpful? RCGP would be a good place to start. I think as they stand the Gps would glace & go , not primary care - I wonder if we could extract the “can do in primary care” bits? (which i appreciate is not as simple as it sounds!) Could they go on microguide? | These pathways are for children presenting to hospital. Although they can be used as the basis for primary care pathways, this would need to be formally done by a group tasked to produce these for primary care clinicians. I agree that the RCGP would be a good place to start. |
| Speciality Group | General |  | A further supportive opinion below. I would also add a minor thing that you have referred to PAUs, many places would call them a CAU and there are probably other acronyms I know nothing about | Amended |
| Speciality Group | General |  | Overall I think they're great - they're user friendly, short enough to be useful and to the point enough to guide immediate clinical practice. I'm sure these pathways will have a very beneficial impact. | No response required |
| Speciality Group | General |  | I'd also be interested to know where these are projected to lie politically between NICE guidance and local guidelines etc. | RESPONSE: these are aligned with NICE guidance and signposts to them where appropriate. However, they cover topics such as antimicrobial stewardship and ambulation that are not covered in NICE or other national guidelines. They will also signpost readers to national empirical AB prescribing recommendations produced by UK-PAS group. They will be used as the basis of teaching. |
| Speciality Group | General |  | Consider using admission unit / ward instead of ssu/Pau as some don’t know what they mean or use a key below. | AGREE - have removed these terms from the title and simply refer to "Hospital". Have changed ED / SSU / PAU to "ED or assessment unit " |
| Speciality Group | General |  | For those units able to consider OPAT – consider giving a card with antibiotic times and red flags for readmission / reassessment | Comment - this is part of the "Ensure robust clinical governance systems and documentation in place for children being ambulated" |
| Speciality Group | General |  | I think they are really nice.  I like the format and flow.  I think they are complimentary to the ENTUK guidelines which are much briefer.  I think they overlap with the mastoiditis and orbital cellulitis guidance, but they don’t appear to be contradictory as far as I could see. | No response required |
| Consultation | General |  | Unclear what the aim of these are. Most services already have clear guidelines- often through multiple discussions through clinical governance meetings from local audit/ local differential diagnosis and have instigated ambulatory care. Is the aim to replace these? If it is to ensure OPAT occurs more swiftly then this should be made clear. | RESPONSE: the aim of these is to promote antimicrobial stewardship and ambulation, as well as optimal infection management. Will make this clear on the webpage on which these pathways will be hosted. Although a tertiary centre such as Oxford may have such pathways, this is not the case for the majority of hospitals in the UK. |
| Consultation | General |  | Most paediatric units see patients up to 16. | Response: there is variation with many units offering the option for young people aged 16-18 years to be managed within a paediatric service or an adult service. RCPCH guidance is up to 18 years for patients being managed within paediatric services. |
| Consultation | General |  | No mention of antipyretics in order to facilitate clinical assessment | Response: not sure how relevant this is. Giving children analgesia and antipyretics is common practice in the UK, we are not proposing that this practice changes. |
| Consultation | General |  | Main comment would be that they just look too complex as currently formatted. | Formatting will be optimised when converted to digital format |
| Consultation | General |  | I wonder if they could have the flow-chart for the most likely/ common management pathways / important features and have the rest as some sort of background links if needed. | Formatting will be optimised when converted to digital format including hyperlinks. |
| Consultation | General |  | Generally the layout is easy to understand and follow, the colours are great but red does not print well, maybe the boxes could be white with colour coded lines in case people may decide to print. | Formatting will be optimised when converted to digital format. These are to be used on a computer, handheld device or mobile phone. |
| Consultation | General | Duration of antibiotics | Re duration of antibiotics I would suggest to give a more prescriptive duration for uncomplicated disease (I understand this may be a bit political and may go against some of the NICE guidelines but this is a document for paediatricians to paediatricians and I think we should advocate for the children) | Response: Duration will be provided and will be aligned with UK-PAS national empirical Ab guidelines. |
| Consultation | General | Dose of antibiotics | I would add a comment on adequate dose in each pathway when enteral therapy is recommended or when failure of oral treatment in mentioned. | Response: have amended to reflect this. |
| Consultation | General |  | In all of the durations I would specify for uncomplicated infection duration is xx. ID/micro advice for  complicated infections | Response: Duration will be provided and will be aligned with UK-PAS national empirical Ab guidelines. Have added "Seek ID/micro advice if complex infection" to all relevant pathways |
| Consultation | General |  | Will there statements re how these guideline should be used alongside existing national guidelines such as NICE Feverish Illness/ NICE SEPSIS/NICE Meningococcal -  is it anticipated that we use these instead? Or will we have guidance on how to practically work with all of them? | RESPONSE: these are aligned with NICE guidance and signposts to them where appropriate. However, they cover topics such as antimicrobial stewardship and ambulation that are not covered in NICE or other national guidelines. They will also signpost readers to national empirical AB prescribing recommendations produced by UK-PAS group. They will be used as the basis of teaching. |
| Consultation | Lymphadenitis | Differentials | Bottom of list is cut off | Formatting will be optimised when converted to digital format |
| Consultation | Lymphadenitis | Differentials | Violet box at bottom cut off at point 5 | Formatting will be optimised when converted to digital format |
| Consultation | Lymphadenitis | Differentials | Mumps “re” parotitis. What is the ‘re’. Does this box go off the bottom of the page? I think most of these are NOT actually in the differential for a “unilateral, red, hot, tender neck lump” but more for generalised LN | AGREE - amended to "Mumps - consider if parotid swelling" . Most of the differentials can present as a unilateral neck swelling. |
| Consultation | Lymphadenitis | Management>mild infection | “source control” perhaps ambiguous. How about “No improvement despite >48hrs of adequate oral Abx (and consider USS to look for abscess)”. It doesn’t matter whether the Abx were prescribe | AGREE - "Source control" changed to "Incision and drainage" |
| Consultation | Lymphadenitis | Differentials | Differentials: does not all fit in the box! Box with red flags is missing | Formatting will be optimised when converted to digital format |
| Consultation | Lymphadenitis | Investigations | If retropharyngeal abscess is suspected could have lateral neck X ray (quicker, safer and easier to obtain late at night and weekend pre CT) | RESPONSE from ENT UK "A lateral neck X-ray does not help understand the true site and extent of infection prior to drainage so I think I would stick to CT. We only really undertake Lateral necks for foreign bodies." |
| Consultation | Lymphadenitis | Management | Management again I would refer to adequate dose/kg here too when referring to failure of oral | Amended |
| Consultation | Lymphadenitis | Formatting | Box cut off at the bottom | Formatting will be optimised when converted to digital format |
| Speciality Group | Lymphadenitis |  | The grey box is cut off. | Formatting will be optimised when converted to digital format |
| Consultation | Meningitis > 3 mths | Management>Antibiotic Duration | *H influenzae* – take out (capsulate), as the serotyping result, incl whether it is an encapsulated strain would often be delayed (as organism gets sent to reference laboratory) and it would not change clinical management in the acute setting. | Agree - amended |
| Consultation | Meningitis > 3 mths | General | To consider adding the point about notifying local health protection team for cases of suspected meningitis for household/close contact chemoprophylaxis | Agree - amended |
| Consultation | Meningitis > 3 mths |  | Mention ‘early/acute complications’ rather than just complications of meningitis occurring within first 3 days.  Appreciate this is acute pathway but worth mentioning following for those with confirmed or suspected meningitis (concern that it may be lost if ambulated early) | Agree - amended re "Early/acute complications…" Have also added "Ensure out-patient follow-up in place to monitor for long term effects of bacterial meningitis as per national guidance." |
| Consultation | Meningitis > 3 mths | Formatting | Arrows misaligned between yellow and orange boxes | Amended and formatting will be optimised when converted to digital format |
| Speciality Group | Orbital cellulitis |  | Add Consider MRI to periorbital / orbital cellulitis pathway | RESPONSE from ENT-UK: MRI is the exception rather than the rule. CT scanning is the first line management for sinusitis complication |
| Speciality Group | Orbital cellulitis |  | in the orbital cellulitis pathway the box on the far right of the first page has preseptal cellulitis as its heading – should it be periorbital as the box below is headed orbital? I thought preseptal cellulitis was a subdivision of periorbital cellulitis I think in the ENT UK guideline we had gone for pre and post septal cellulitis. The terminology can be confusing though. I’m not sure the term periorbital is helpful. | AMENDED TO PRESEPTAL AND POSTSEPTAL (ORBITAL) CELLULITIS |
| Consultation | Orbital cellulitis | General | As no specific antibiotic recommended, perhaps add ; in accordance with local guidelines’ – which is what you have referred to in other pathways ? | RESPONSE: in addition to local guidelines, readers will be signposted to the national guidance produced by UK-PAS |
| Consultation | Orbital cellulitis | Management | As no specific antibiotic recommended, perhaps add ; in accordance with local guidelines’ – in keeping with what is suggested for other pathways ? | RESPONSE: in addition to local guidelines, readers will be signposted to the national guidance produced by UK-PAS |
| Consultation | Orbital cellulitis | Management>pre-septal | Topical Decongestants – should there be an age recommendation for this – unlikely to helpful in younger children | RESPONSE from ENT-UK (Andrea Burgess)- topical decongestants are commonly used, even in younger children. No concerns about their short term use. The BMJ in 2019 warned against usage in children younger than 6 years - the pathway has been amended to reflect this to "older children > 6 years" " |
| Consultation | Orbital cellulitis | Red flags | Might be worth using ‘preseptal (periorbital) and postseptal (orbital)’ to avoid confusion.  Chemosis is supposed to make orbital cellulitis more likely | Amended to preseptal and postseptal (orbital) as per consultation comments. Chemosis of no added value ot the other symptoms listed in the table |
| Consultation | Orbital cellulitis | Investigations | “endonasal”. Is that the same as ‘nasal’ or does it need clarifying | Amended for clarification: "If nasal endoscopy performed by ENT team, collect sinus swab (endonasal swab)" |
| Consultation | Orbital cellulitis |  | Orbital cellulitis is one of the danger signs of a severe dental infection. In the 'PRE-SEPTAL AND POSTSEPTAL (ORBITAL) CELLULITIS PATHWAY' differential diagnosis it suggests that dental infections should only be considered if the swelling is at cheek level.  If a child has orbital cellulitis, it would be prudent for any clinician to assume that it could be from a dental source (especially an upper canine tooth) and if decayed teeth are identified by visual inspection in the mouth then oral surgeons/paediatric dentists should be invited to assist with the diagnosis and provision of an operative procedure to remove the source of the infection alongside antibiotic therapy as an important part of the treatment plan. | RESPONSE: in children, it is far more common for the source of infection to be the sinus than from a dental infection. It is probably not appropriate for the default to be that the source of infection is of dental origin. This is why we have provided guidance about feature that make an dental origin more likely - have amended to " If swelling at cheek level, consider dental origin and conduct visual inspection for decayed teeth (esp. upper canine teeth). Consider dental/maxfax review and/or odontogram." |
| Consultation | Orbital cellulitis | Investigations | Aptima swabs in neonatal and adolescents if Chlamydia/GC suspected | RESPONSE - this is a pathway for preseptal and postseptal cellulitis. Ophthalmia neontorum is on the differential list but this pathway is not a pathway for the management of the neonate with suspected ophthamlia neonatorum |
| Consultation | Orbital cellulitis | Management>Preseptal | For the moderate/ severe preseptal cellulitis, ENT and ophthalmology review should be asked for (not included in this section draft currently, rather only recommended in the orbital cellulitis section) | Agree - added to amber management box "ENT and ophthalmology review if moderate / severe pre-septal cellulitis." |
| Speciality Group | Orbital cellulitis |  | Differentials should include late presentation of a rhabdomyosarcoma/retinoblastoma | Added to differential list |
| Speciality Group | Orbital cellulitis |  | Add Usually to unilateral - having said that I can’t remember seeing a bilateral case but I’m sure it can happen | Bilateral eyelid swelling / oedema would make a diagnosis of periorbital or orbital cellulitis extremely unlikely. Unable to find any case reports of this in children. |
| Speciality Group | Orbital cellulitis |  | Dacrocystitis | Amended |
| Speciality Group | Orbital cellulitis |  | Didn’t know >9 associated with increased severity - most of the ones I’ve seen have been <5 | RESPONSE: Agree. Looking at the literature, no clear data to support age cutoff. 10 year case series from Portugal (https://pubmed.ncbi.nlm.nih.gov/30772617/) - median age of preseptal cellulitis 5.9 years (n=94), median age orbital cellulitis 6.8 years (n=24). Age recommendations removed. |
| Speciality Group | Orbital cellulitis |  | I wouldn’t do investigations in any preseptal (says mild), but would if I thought it was orbital. | Good point - amended to advice no bloods if mild or moderate infection as these are unlikely to yield anything useful. Just if severe preseptal cellulitis (where we think possible orbital cellulitis or systemically unwell) have we recommended that blood tests are performed. |
| Speciality Group | Orbital cellulitis |  | They talk about mild preseptal and significant periorbital swelling in management without defining them | AGREE - increased disease severity is now clearly defined. MILD and MODERATE is everything that isn't INCREASDED disease severity |
| Speciality Group | Orbital cellulitis |  | Don’t mention drainage of sinuses | Orbital and sinus drainage is indicated if radiological evidence of orbital collection this is either totally endoscopically or part external / part endoscopic depending on surgeons experience. Smaller collections may be treated with IV antibiotics. Severe pre-septal cellulitis without evidence of collection would generally be treated with ongoing Abs and rescanning. One may also consider sinus drainage to remove reservoir of infection if poor response rate. Added following text to orange management box "Consider sinus drainage to remove reservoir of infection if poor response rate to IVAbx and collection on imaging." |
| Speciality Group | Orbital cellulitis |  | agree if need admitting should be under paeds | Have amended |
| Speciality Group | Orbital cellulitis |  | Not quite sure whether COVID has made any difference | No data published on the frequency of presentations with preseptal or orbital cellulitis and will not change the presentation or management. |
| Speciality Group | Orbital cellulitis |  | I am uncomfortable about the statement about younger children - not really sure if its true or not - but the message is that younger children don’t get severe disease could lead to severe infection being over looked, in an age group who are difficult to examine and who are very good at not looking unwell until they aren't | RESPONSE: Agree. Looking at the literature, no clear data to support age cutoff. 10 year case series from Portugal (https://pubmed.ncbi.nlm.nih.gov/30772617/) - median age of preseptal cellulitis 5.9 years (n=94), median age orbital cellulitis 6.8 years (n=24). Age recommendations removed. |
| Speciality Group | Orbital cellulitis |  | The signs don’t mention hypoglobus as a sign of a collection, and therefore an indication for scan - which leads to a change in management. there is not much in the literature - but for me is far more common in kids than proptosis - its mentioned in the attached 2016 paper, and apparent in case 1 in the 2012 paper | Response from ENT-UK: in infection, proptosis is the most obvious sign, Hypoglobus (downward displaced eye) associated with proptosis ( particularly in large collections) would definitely be a sign of collection. Hypoglobus alone would also warrant scan but raises concerns of other conditions such as infraorbital fractures, neoplasms or silent sinus syndrome. Added to table as should warrant imaging. |
| Speciality Group | Orbital cellulitis |  | It always difficult to write a guide in this complex area, where often three specialties need to do their bit - so I think co management with paediatrics could be mentioned as well, but this may not always happen if the unit is isolated | Have added "If admitted, should be under general paediatric team or paediatric ENT team. " |
| Speciality Group | Orbital cellulitis |  | Seen a few Guidelines for this before so not sure where this fits in the hierarchy and exactly who is aimed at? It is probably me but not that familiar with BSAC. Not sure what is COVID different about this? | This guideline is not influenced by COVID> The focus of this pathway is clear information promoting antimicrobial stewardship, ambulation where possible and optimal infection management. BSAC is the British Society for Antimicrobial Chemotherapy. |
| Speciality Group | Orbital cellulitis |  | Agree with previous comments especially about age. Could change Proptosis to Proptosis Globe displacement. Drainage mentioned in orange box | See comment above. |
| Speciality Group | Orbital cellulitis |  | Also not exactly sure what abbreviations SSU and PAU stand for - there may be different abbreviations used across different Trusts | Amended to "ED or assessment unit" |
| Speciality Group | Orbital cellulitis |  | Not sure what ambulation on IVAb’s means exactly? Is this home on IVAb’s? - found it a bit confusing | This guidance is aimed primarily for front line paediatric staff who are well versed with ambulation on IVAbx |
| Speciality Group | Orbital cellulitis |  | Would be helpful if written safety netting info referred to was appended was appended? | This pathway will be hosted online and the safety netting sheet will be hyperlinked directly from the pathway |
| Consultation | Pneumonia | General | I suspect that there will be a lot of children who may end up in the moderate severity category (due to fever and some respiratory distress), with a viral aetiology and will end up getting antibiotics empirically. Would consider getting investigations in moderate severe category i.e. viral swabs for viral respiratory testing and FBC/CRP and adopt a watch and review with result approach. | RESPONSE: although using investigations and adopting a watch and wait approach for children in the moderate group seems like a highly sensible approach, there is no evidence from real world studies to support that this approach reduced antimicrobial prescribing (as the majority of these children are discharged from ED). The BPRS have made it clear in their guidance that there is no role for investigations in this moderate groups (for the reasons stated previously) - until evidence emergences about the role of POC diagnostics in this group, we are unable to make the recommendation that you suggest. |
| Consultation | Pneumonia | Management | If you had definite lobar pneumonia on CXR, should you give Abx even if well/mild? We would not standardly perform CXR and manage the child clinically and would only suggest if lack of improvement  How is RDS defined | RESPONSE: there is no answer to this question as we do not routinely X-ray these children. It is the views of the steering group that children with mild symptoms (with no respiratory distress) do not need to start Abx. The approach we are promoting in such cases is robust safety netting. |
| Consultation | Pneumonia | Assessing severity | CAP I would stress in the assessing severity that fever needs to be present otherwise lots of viral wheeze will enter in the severe category… Difficult to convey I know it is in the diagnosis already but would stress here too. | RESPONSE: have added "Presence of fever is an essential feature in CAP" to assessment box |
| Consultation | Pneumonia | Management | Re management: empyema present: Drainage to be considered depending on the size of the collection (if >2 cm is it necessary to wait for 48 hrs before putting a chest drain ???)/ When adequate abx mentioned add adequate dose per kg | Response from BPRS - depth is a guide but highly variable in terms of measurement but should not mandate surgical management. Management is based on clinical decision making. |
| Consultation | Pneumonia | Investigations | Consider Legionella & Mycoplasma | RESPONSE: Legionella testing is not a common respiratory pathogen in children and should not be routinely tested. Mycoplasma testing (acute/convalescent serology and/or PCR) has little role in acute management as the decision to start a macrolide is based on local/national empirical guidelines and results of convalescent serology are often back after the acute illness has improved. |
| Consultation | Pneumonia | Management | Source isolation and IPC risk assessment for bed placement if respiratory viral infection/influenza suspected | RESPONSE: the focus of these pathways is antimicrobial stewardship and ambulation, as well as optimal infection management. All hospitals have infection control recommendations for children requiring source isolation - this is beyond the scope of these pathways. |
| Speciality Group | Tonsillitis |  | Instead of LFTs of abdo pain, enquire about abdo pain, perform LFTs, check for hepatosplenomegaly | Amended |
| Speciality Group | Tonsillitis |  | I couldn’t see a line about draining a quinsy (older teenagers for example). | RESPONSE: Is already present "If peritonsillar abscess (quinsy), ENT team may decide on early aspiration in older children." |
| Consultation | Tonsillitis | Investigations>Evaluate severity | As for AOM - Moderate severity – seems to suggest all children with tonsillitis and fever are moderately severe and so have bloods taken and receive IV antibiotics. Would be helpful to give more detail in this differentiation – to prevent a lot of children getting IV antibiotics. | RESPONSE: definitions of mild and moderate severity amended as follows "MILD = systemically well or fever but haemodynamically stable. MODERATE = systemically unwell including fever AND persisting tachycardia / tachypnoea" |
| Consultation | Tonsillitis | Investigations | See previous comment in AOM guideline about definition of moderate. Lots of children with fever have concurrent tachycardia and tachypnoea but are not unwell. No mention of antipyretics | RESPONSE: definitions of mild and moderate severity amended as follows "MILD = systemically well or fever but haemodynamically stable. MODERATE = systemically unwell including fever AND persisting tachycardia / tachypnoea". Analgesia with paracetamol and ibuprofen is suggested. |
| Consultation | Tonsillitis | Management | Why not choose FeverPAIN score or centor score, otherwise people might try to use both. Are both/either validated for young children? We could consider the RCH Melbourne approach instead | RESPONSE: NICE guidance is feverpain or Centaur score. People tend to use one or the other. |
| Consultation | Tonsillitis | Management | oral antibiotics for moderate infection if tolerated. Dose as well here | RESPONSE: amended as follows "If no improvement despite ≥72 hours of adequate oral Abx (check dose and adherence" |
| Consultation | Tonsillitis | Investigations | Rapid antigen tests for detection of group A streptococcal antigen on a throat swab are not recommended, Travel and vaccination history | RESPONSE: agree that rapid Gp A strep test is not recommended in the UK by NICE. It is assumed that a full history is taken including travel history and vaccine history. |
| Consultation | Tonsillitis | Investigations | EBV serology preferred to Monospot test in children who are immunocompromised at any age | RESPONSE: it is not clear to me why the Monospot (hererophile Abs) is more affected than EBV serology in the immunocompromised host - neither are optimal as they rely on antibody responses to EBV (https://www.ncbi.nlm.nih.gov/pmc/articles/PMC3782265/) |
| Consultation | UTI | Management | Some units use urine pads, not bags: so perhaps add pads to comment about not sending bag specimens to lab | Added |
| Consultation | UTI | Management | Would you consider adding record urine dip ? – e.g. young child with fever, culture mixed growth, can be useful to know if leu/nit pos or not | Added |
| Consultation | UTI | Investigations>toilet trained children | Would be worth elaborating what a correctly performed clean voided urine sample is-this is very subjective | RESPONSE: have added the following sentence after "For toilet trained children send correctly performed clean voided urine samples" - "Provide clear instructions to parents/carers" and have hyperlinked to GOSH info sheet for parents |
| Consultation | UTI | Investigations> infants not potty trained | Some units use pads, not bags, suggest add pads to comment about not sending bag specimens to lab | Added |
| Consultation | UTI | Investigations> infants not potty trained | Suggest add record urine dip – e.g. young child with fever, culture mixed growth, can be useful to know if leu/nit pos or not | Added |
| Consultation | UTI | Investigations | TUBC – took me a while to work this one out. It might be better to have something that does not require people to look up in abbreviations e.g. in out catheter | RESPONSE- amended to "in and out catheterisation" |
| Consultation | UTI | Investigations | a) Here you suggest TUBC or SPA but in the ‘fever without source’ guide, you only suggest TUBC. I’d guess that SPA is usually second line for most people, probably has a slightly higher complication rate, and if recommending it you should probably mention that it should ideally be done after bladder USS b) For toilet trained children, they should aim for a MID-STREAM urine. c) For non-toilet trained children who are well, we are usually happy with a clean catch urine (two samples even better) – especially in boys, especially with the quick wee method. | RESPONSE - amended to "midstream clean catch urine sample" |
| Consultation | UTI | Management | Is there an age group where you would NOT switch to orals. E.g. under 6wks? | RESPONSE - no lower age limit for oral switch option |
| Consultation | UTI | Box with *** | Concomitant meningitis is uncommon OVER 5 WEEKS – but the guideline suggests doing LP if under 4 wks. Other guidelines have suggested if under 6weeks. Could be more consistent | RESPONSE: have amended to reflect the literature "Concomitant UTI and meningitis is uncommon in infants and children over 4 weeks of age" |
| Consultation | UTI | Investigations | : investigations: i) I would add for the clean catch in the non toilet trained babies something about cleaning the perineum first.  LP if <1 month …. I would be consistent with previous recommendation and rephrase meningitis is rate in the presence of proven UTI ,can consider LP if signs of meningitis or red flags or similar this is somewhere in the back of the page and not v visible. ? add a box with suggestions of how to collect a urine sample in the non toilet trained babies (15 min after a feed, massaging bladder, etc) | RESPONSE: the steering group have not recommended clean catch urine samples in non toilet trained children with presumed pyelonephritis. LP recommendations reworded "Empirical lumbar puncture (LP) if <28 days with presumed PN/upper UTI4-6 or clinical concerns for bacterial meningitis – check for contraindications to LP" |
| Consultation | UTI | Management | stress the need to collect x2 urine samples before antibiotics if possible. Duration 7 days | RESPONSE: duration recommendations amended in line with UK-PAS empirical Ab guidelines "Total duration of treatment (IV+oral) = 7 days (10 days if bacteraemia)". NICE do not recommend 2 urine samples before commencing Abs - we have stressed the importance of a properly collected urine sample prior to commencement of Abs |
| Consultation | UTI | Investigations | ??TUBC | Amended to "in out catheter" |

-End-
